# Orexin Receptor Antagonism Improves Sleep Quality and Mitigates Lipopolysaccharide‐Induced Inflammatory Responses in a Mouse Model

**DOI:** 10.1096/fj.202502960R

**Published:** 2026-01-09

**Authors:** Dai Horiuchi, Yoko Irukayama‐Tomobe, Jun‐Dal Kim, Yoshitoshi Kasuya, Tsuyoshi Nemoto, Takuji Suzuki, Yoshimi Nakagawa, Koichiro Tatsumi

**Affiliations:** ^1^ Department of Respirology, Graduate School of Medicine Chiba University Chiba Japan; ^2^ Division of Complex Biosystem Research, Department of Research and Development, Institute of National Medicine University of Toyama Toyama Japan; ^3^ Life Science Center for Survival Dynamics, Tsukuba Advanced Research Alliance (TARA) University of Tsukuba Ibaraki Japan; ^4^ Amed‐Crest Japan Agency for Medical Research and Development Tokyo Japan; ^5^ Department of Molecular and Systems Pharmacology, Faculty of Pharmacy Juntendo University Chiba Japan; ^6^ Chiba University Hospital Chiba Japan; ^7^ International Institute for Integrative Sleep Medicine (IIIS) University of Tsukuba Tsukuba Japan

**Keywords:** cytokines, daridorexant, inflammation, lipopolysaccharide, sleep/wakefulness disorders

## Abstract

Alterations in the immune system, stemming from sleep/wakefulness disorders, increase the risk of inflammatory pathologies. Orexin, a hypothalamic neuropeptide, regulates sleep and wakefulness. However, the role of orexin in inflammatory responses—whether it is protective or pathological—is still unclear. In this study, our aim was to elucidate the role of orexin in sleep and inflammatory states through the examination of a lipopolysaccharide (LPS)‐induced systemic inflammatory model and the effects of daridorexant, a dual orexin receptor antagonist. Intraperitoneal LPS administration significantly decreased rapid eye‐movement (REM) sleep and wakefulness while increasing non‐REM sleep. Pretreatment with daridorexant enhanced REM sleep recovery in LPS‐induced systemic inflammation, evidenced by extended duration and increased episode frequency. Transcriptomic profiling demonstrated a rise in the expression of pro‐inflammatory cytokines (Cxcl1, Ccl2, Ccl7, and Tnf) within the hypothalamus of LPS‐challenged mice, which was mitigated by daridorexant administration. In addition, daridorexant mitigated LPS‐induced acute lung inflammation. These findings suggest that by reducing pro‐inflammatory cytokine expression, the inhibition of orexin activity mitigates the lethargy associated with systemic inflammation, while also improving sleep quality. This study explores the potential of orexin receptor antagonists as strategic options for inflammatory pathologies and their associated sleep disorders.

## Graphical abstract

Inhibiting orexin improves sleep quality and mitigates inflammation of LPS‐induced mice.

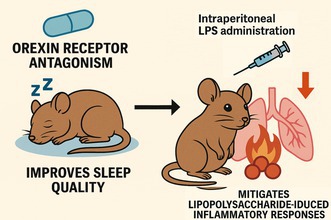

## 1 Introduction

Sleep is essential for managing stress, improving memory, and regulating hormones that control appetite and metabolism; furthermore, high sleep quality is crucial for reducing systemic inflammation and ensuring neurocognitive health [1]. The association between sleep disorders and inflammation is well‐documented. For instance, disrupting sleep often triggers acute inflammation [2], and sleep deprivation is known to elevate levels of pro‐inflammatory cytokines [3].

Orexins (also known as hypocretins) are hypothalamic neuropeptides crucial for maintaining wakefulness. Mice deficient in orexin/ataxin‐3, characterized by the genetic ablation of orexin‐producing neurons, show a faster recovery from LPS‐induced sickness than wild‐type mice [4, 5]. Consistent with its role, orexin neuronal activity is suppressed during acute systemic inflammation [6], and cerebrospinal fluid (CSF) orexin levels are reduced in patients with sepsis [7]. This reduction in orexin activity consequently facilitates sleep induction and limits physical activity. It remains unclear, however, whether these responses lead to pathological outcomes (e.g., impaired wakefulness, increased lethargy, and heightened inflammation) or protective adaptations (e.g., extended sleep duration that facilitates recovery and attenuates inflammatory burden). Therefore, elucidating the mechanism underlying this bidirectional role of orexin activity is of interest.

Importantly, most previous studies investigating the link between orexin and inflammation have relied on constitutive knockout models, in which orexin signaling is absent throughout development. While such approaches have provided important insights, they cannot capture the temporal and reversible regulation of orexin signaling that would be more relevant to clinical contexts. Thus, the novelty of the present study lies in the use of a pharmacological approach with daridorexant, which allows acute, reversible inhibition of orexin signaling. This enables a more clinically translatable evaluation of how orexin activity influences sleep and inflammatory responses.

Inflammation dysregulates sleep–wake activity. There are two primary types of sleep: nonrapid eye‐movement (NREM) sleep and REM sleep. Acute mild inflammation inhibits REM sleep, while severe inflammation suppresses both NREM and REM sleep [1]. Lipopolysaccharide (LPS), a major component of the cell wall in gram‐negative bacteria, acts as a cytokine inducer and is widely used in systemic inflammation models. In LPS‐injected mice, REM sleep is attenuated, and NREM sleep is rapidly induced [8]. Locally produced inflammatory signals during acute inflammation alter the activity of the hypothalamic homeostatic system. Among the key neuronal populations involved in regulating arousal and physical activity are orexin (hypocretin)‐expressing neurons in the lateral hypothalamic area. Orexin modulates arousal in response to metabolic needs, stress, and reward stimuli, playing a crucial role in regulating REM sleep [9]. Peripherally administered LPS reduces the activity of orexin neurons in the lateral hypothalamic area [6], likely contributing to LPS‐induced anorexia. Given orexin's central role in the arousal system, its inhibition by LPS may underlie the lethargy and inactivity observed during systemic inflammation. To further investigate the peripheral consequences of systemic inflammation, we collected bronchoalveolar lavage fluid (BALF) to directly evaluate pulmonary inflammation. BALF analysis is a well‐established method to assess leukocyte infiltration and cytokine/chemokine levels in the airways, and it has been widely applied in preclinical models of lung injury and inflammation [10, 11]. This approach complemented hypothalamic analysis and provided insight into both central and peripheral inflammatory responses.

Orexin receptor antagonists have recently gained attention as therapeutic agents for insomnia. Daridorexant, an orally administered dual orexin receptor antagonist, has been approved for the treatment of insomnia [12]. In this study, we aimed to address the gap in understanding regarding whether reduced orexin activity during systemic inflammation is pathological or protective by evaluating the effects of daridorexant administration on sleep regulation and LPS‐induced systemic inflammation in mice.

## 2 Materials and Methods

### 2.1 Animals

All animal procedures were approved by the Review Board for Animal Experiments of Chiba University and were conducted in compliance with the guidelines of the Animal Research Committee of the Laboratory Animal Center, Graduate School of Medicine, Chiba University (approval number 4–47). Male C57BL/6J mice, aged 8–15 weeks, were obtained from Claire Japan Inc. (Shizuoka, Japan). The mice were housed in an animal experimental facility at Chiba University under specific pathogen‐free conditions in plastic cages at 23°C ± 1°C with a 12 h light/dark cycle. They had unrestricted access to food and drinking water. All experimental procedures adhered to the Animal Research Reporting of In Vivo Experiments (ARRIVE) guidelines.

### 2.2 LPS and Daridorexant Administration to Mice

LPS, derived from *Escherichia coli* 055: B5 (Sigma‐Aldrich, MO, USA), was dissolved in 0.9% sodium chloride saline solution (Otsuka, Tokyo, Japan). Systemic inflammation was induced in mice through the peritoneal injection of LPS at a dose of 2.5 mg/kg, as previously reported [8].

Daridorexant (Idorsia Co. Ltd., Basel, Switzerland) was suspended in 0.5% (w/v) methyl cellulose 400 solution and sterilized (Fujifilm, Tokyo, Japan). The mice received oral administration of daridorexant‐HCl (108 mg/kg, equivalent to 100 mg/kg free base) or vehicle for three consecutive days before LPS injection. The mice were randomly assigned to four groups: LPS + vehicle (LPS group), LPS + Daridorexant group, saline + vehicle (Vehicle group), and saline + daridorexant (Daridorexant group).

### 2.3 Experimental Design and Timeline

**FIGURE 1:**
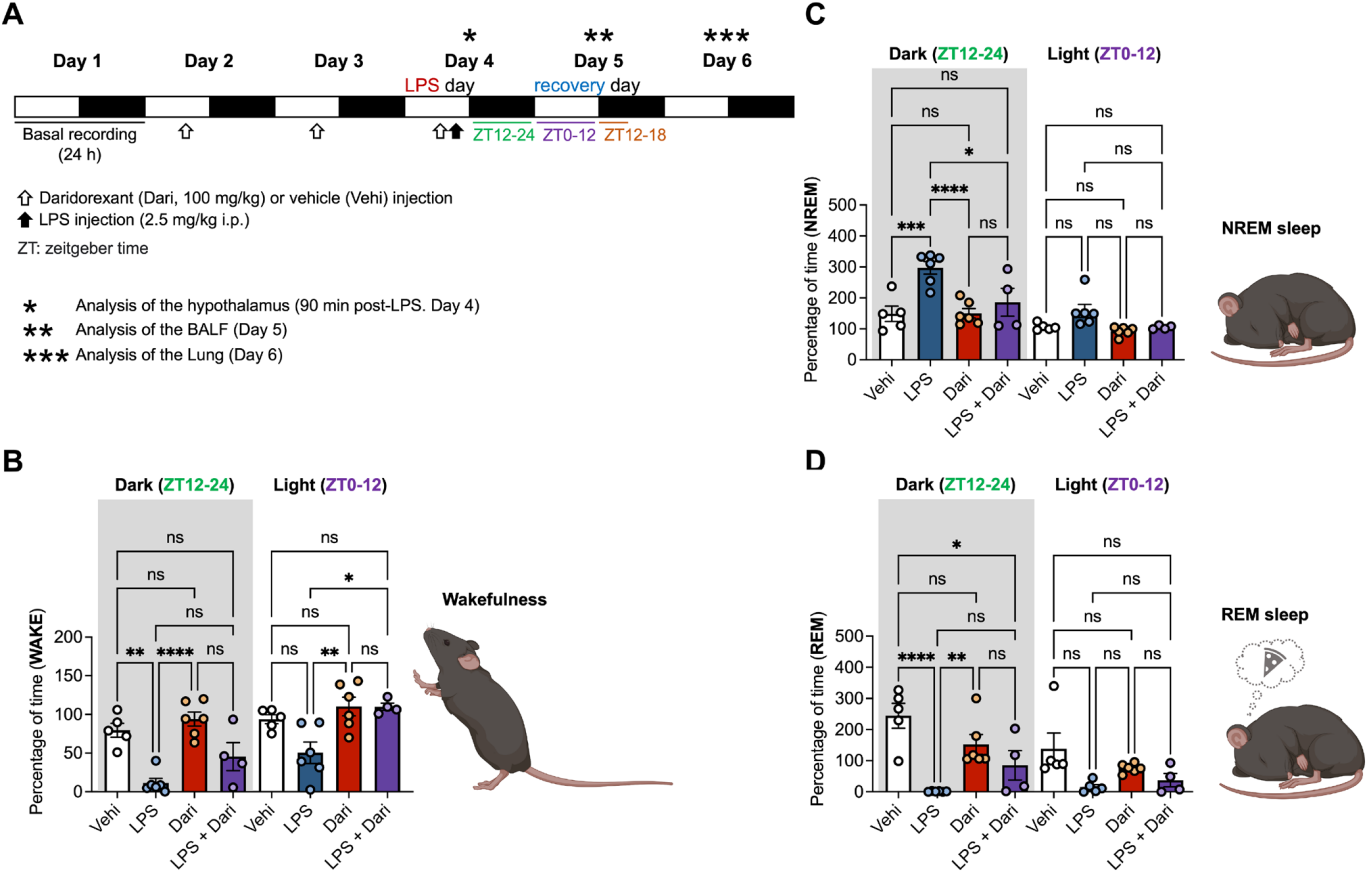
Effects of daridorexant pretreatment on the sleep architecture in LPS‐induced inflammatory mice. (A) Schematic representation of the experimental protocol across six consecutive days. Day 1 (baseline): EEG/EMG baseline recordings. Days 2–3 (drug days): Oral administration of daridorexant or vehicle at ZT7.5. Day 4 (LPS day): Oral administration of daridorexant or vehicle at ZT7.5, followed by intraperitoneal injection of LPS or saline at ZT8. A subset of mice was sacrificed 90 min after LPS or saline injection on Day 4 for hypothalamic RNA analysis. Day 5 (recovery day): Continuous EEG/EMG recording and BALF collection 24 h after LPS injection. Day 6: Lung samples were perfused, fixed, and collected for histological evaluation. (B–D) Effects of LPS and/or daridorexant administration on the relative percentages of wakefulness (B), NREM sleep (C), and REM sleep (D) during the dark phase (ZT12–24) and light phase (ZT0–12), normalized to the basal recording (intact day). Vehi = Vehicle, Dari = Daridorexant, LPS + Dari = LPS + Daridorexant. Data are presented as mean ± standard error of the mean (*n* = 4–6 per group). *p < 0.05, **p < 0.01, ***p < 0.001, ****p < 0.0001 and ns (not significant). Statistical analysis was performed using one‐way analysis of variance (ANOVA), followed by Tukey's post hoc test for multiple comparisons.

To clarify the experimental design, the complete protocol is illustrated in (Figure 1). EEG/EMG recordings and sample collections were conducted over six consecutive days. Day 1 served as the baseline (intact) day. On Days 2 and 3, mice received oral administration of daridorexant or vehicle at ZT7.5. On Day 4 (LPS day), daridorexant or vehicle was again administered at ZT7.5, followed by an intraperitoneal injection of LPS or saline at ZT8. Day 5 was defined as the recovery day, and on Day 6, lung samples were collected for histological analysis.

For molecular analyses, a subset of mice was sacrificed 90 min after LPS or saline injection on Day 4 to evaluate early transcriptional responses in the hypothalamus. For pulmonary inflammation assessment, BALF was collected 24 h after LPS injection on Day 5. For histopathological examination, the left lung lobe was perfused, fixed, and excised on Day 6.

### 2.4 Electroencephalography/Electromyography Recording

For sleep recordings, two EEG screws and EMG wires were surgically implanted under isoflurane anesthesia. The EEG screws were placed in the skull, and the EMG wires were inserted into the trapezius muscles. The electrode assembly was secured to the skull with dental acrylic resin, as described previously [13].

The mice were housed in experimental cages and allowed to habituate to the recording environment for over 1 week. EEG and EMG signals were amplified and filtered (EEG, 0.5–64 Hz; EMG, 20–200 Hz) and digitized at a sampling rate of 128 Hz. Data were recorded using the Vital Recorder software (Kissei Comtec, Nagano, Japan). EEG/EMG signals were first analyzed automatically in 10 s epochs using Sleep Sign software (Kissei Comtec). The results were manually reviewed and finalized. Sleep stages were classified as wakefulness, NREM sleep, or REM sleep. The percentage of total time spent in each stage was calculated relative to the baseline (Day 1). Data were expressed as a percentage of baseline to account for interindividual variability in absolute sleep/wake parameters and to allow within‐subject comparisons across experimental days.

### 2.5 RNA Preparation and RNA‐Sequencing

To confirm early gene expression, mice were anesthetized by inhalation of isoflurane (Viatris, Tokyo, Japan) 90 min after the administration of LPS or saline, daridorexant, or vehicle and intracardially perfused with ice‐cold diethylpyrocarbonate‐treated phosphate‐buffered saline (PBS) to remove blood cells from the brain and were sacrificed. The brains were quickly dissected to isolate the hypothalamic tissue after removing the cortex, cerebellum, and olfactory bulbs. The hypothalamic tissue was homogenized in ISOGEN (Fujifilm), and total RNA was extracted.

Approximately 500 ng of total RNA was depleted of ribosomal RNA using the NEBNext rRNA Depletion Kit (New England Biolabs, MA, USA) and converted into an Illumina sequencing library with the NEBNext Ultra Directional RNA Library Prep Kit for Illumina (New England Biolabs). Library validation was performed using a Bioanalyzer (Agilent Technologies, CA, USA), and sequencing was carried out on the NextSeq 500 platform (Illumina, CA, USA) with paired‐end 36‐base reads. Sequencing reads were mapped to the mouse reference genome (GRCm38/mm10) and quantified using the CLC Genomics Workbench (version 12.0, QIAGEN, Venlo, Netherlands).

### 2.6 Identification of DEGs and Functional Enrichment Analysis

To identify DEGs between the Vehicle and LPS groups and between the LPS and LPS + Daridorexant groups, read counts were normalized by calculating the number of reads per kilobase per million for each transcript in individual samples using the CLC Genomics Workbench (version 12.0, QIAGEN). DEGs were identified using a fold‐change threshold of −2 to +2 and a false discovery rate of *p* < 0.05. Gene expression differences were visualized through PCA and heat map clustering. Volcano plots were generated to illustrate the significance of changes in gene expression by plotting −log10 (*p* value) and log2 fold change.

Functional enrichment analyses of DEGs were performed using GO (biological process) and KEGG pathway analyses. These analyses were conducted using the Web‐based *Enrichr* suite (http://amp.pharm.mssm.edu/Enrichr) [14, 15, 16].

### 2.7 Quantitative Real‐Time PCR Analysis

Approximately 400 ng of total RNA was reverse transcribed into complementary DNA using ReverTra Ace (TOYOBO, Osaka, Japan) according to the manufacturer's instructions. Quantitative real‐time PCR was performed on a duplex CFX Duet real‐time PCR system (BIO RAD, CA, USA) using SYBR Green KAPA qPCR Master Mix (Loche, Basel, Switzerland). The PCR protocol included an initial denaturation cycle at 95°C for 10 min, followed by 40 cycles of denaturation at 95°C for 15 s and annealing/extension at 60°C for 1 min. Predesigned primers were used to amplify the following genes: *Cxcl1* (Primer Set ID: MA231723, TAKARA, Kyoto, Japan), *Ccl2* (Primer Set ID: MA267402, TAKARA), *Ccl7* (Primer Set ID: MA25976, TAKARA), *Tnf* (Primer Set ID: MA208332, TAKARA), *Gapdh* (Primer Set ID: MA050371, TAKARA), and *Hprt* (forward primer, 5′‐TCCTCCTCAGACCGCTTTT‐3′; reverse primer, 5′‐CCTGGTTCATCATCGCTAATC‐3′). Gene expression levels were normalized to either *Gapdh* or *Hprt* mRNA levels. The relative gene expression was calculated using the ΔΔCt method for all comparative analyses.

### 2.8 BALF Collection and Mouse Cytokine Array

LPS was administered on Day 4 (LPS day), and BALF was collected 24 h later on Day 5 (recovery day). BALF was obtained by flushing the lobes twice with 1 mL of PBS containing phenylmethanesulfonylfluoride. A total of 0.8 mL of BALF was collected per mouse, centrifuged at 500 *g* at 4°C for 3 min, and the supernatants were immediately stored at −80°C for cytokine measurement.

The levels of cytokines and chemokines in BALF were quantified using the Mouse Inflammation Antibody Array I (RayBiotech, Norcross, GA, USA) following the manufacturer's protocol. The mean signal of the positive control spots was assigned an arbitrary value of 10, and the spot signal values for each cytokine/chemokine were expressed as arbitrary units. Data were compared across the four experimental groups.

### 2.9 Pathological Analysis

On Day 6, left lung lobe samples were perfused with PBS, excised, and post‐fixed in 4% paraformaldehyde. The tissues were then immersed in 30% sucrose in PBS until fully saturated and subsequently embedded in Optical Cutting Temperature Compound (O.C.T. Compound, Sakura Finetek, Torrance, CA, USA). The lungs were cryosectioned at a thickness of 5 μm using a cryostat (Leica, Wetzlar, Germany) and stained with hematoxylin and eosin. Microscopic images of the lung sections were captured using a BZ‐800 microscope (KEYENCE Inc., Osaka, Japan). Lung injury was assessed by measuring alveolar wall thickness using semi‐quantitative light microscopy with the hybrid cell count module of the BZ‐X800 analyzer (KEYENCE Inc.). Alveolar wall thickness was expressed as a percentage of the alveolar surface area, calculated by averaging the measurements from four randomly selected fields per section.

### 2.10 Statistical Analysis

**FIGURE 2:**
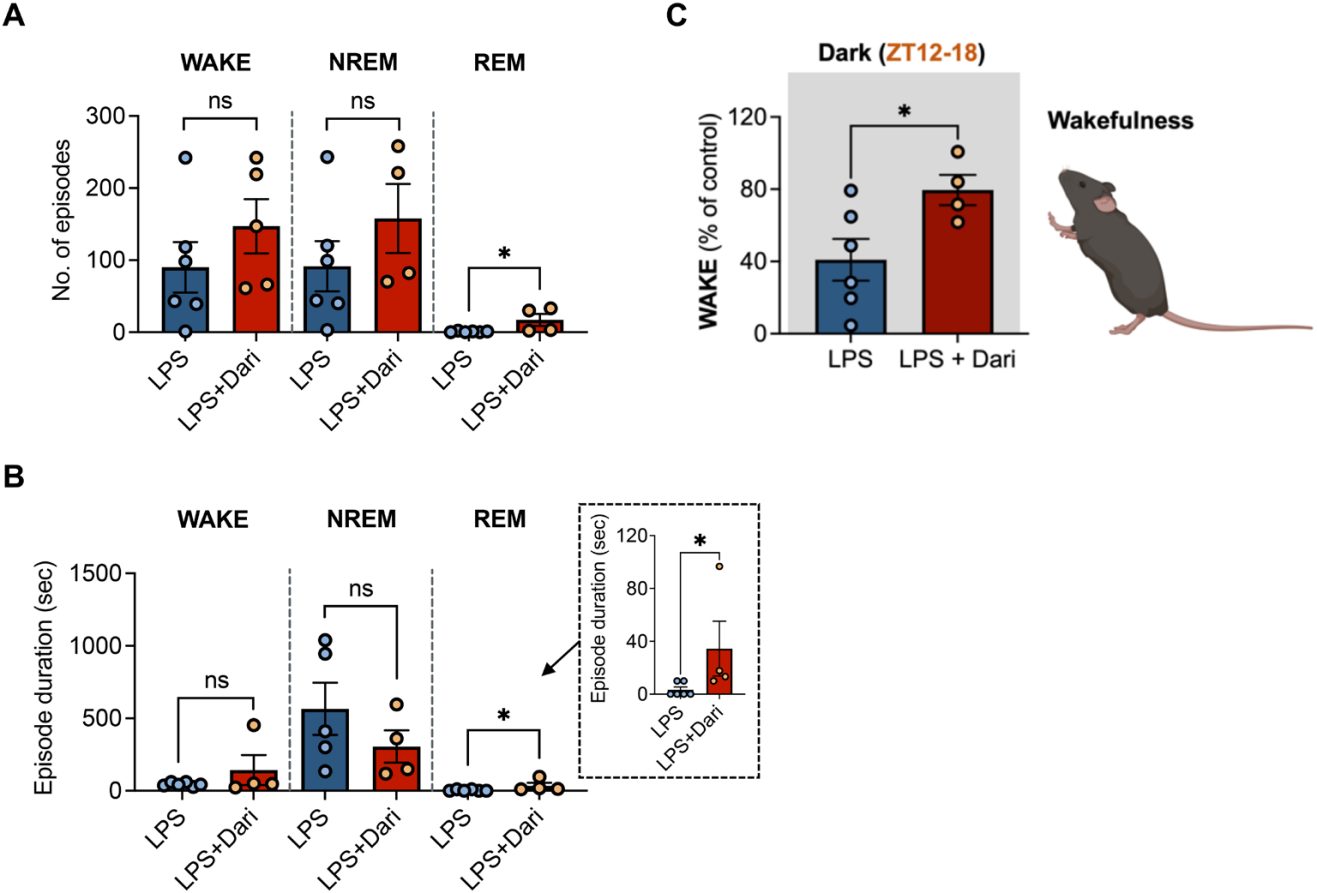
Changes in sleep episodes of LPS‐induced inflammatory mice with orexin receptor antagonism. (A, B) Number of episodes (A) and duration of wakefulness (B), NREM, and REM states during the dark phase (ZT12–24) on Day 4 (LPS Day) in the LPS and LPS + Daridorexant groups. (C) Time spent awake in the dark phase (ZT12–18) on day 5. Data are presented as mean ± standard error of the mean (*n* = 4–6 per group). *p < 0.05, ns (not significant) vs. LPS group. Statistical analyses were performed using unpaired two‐tailed Student's t‐tests.

**FIGURE 3:**
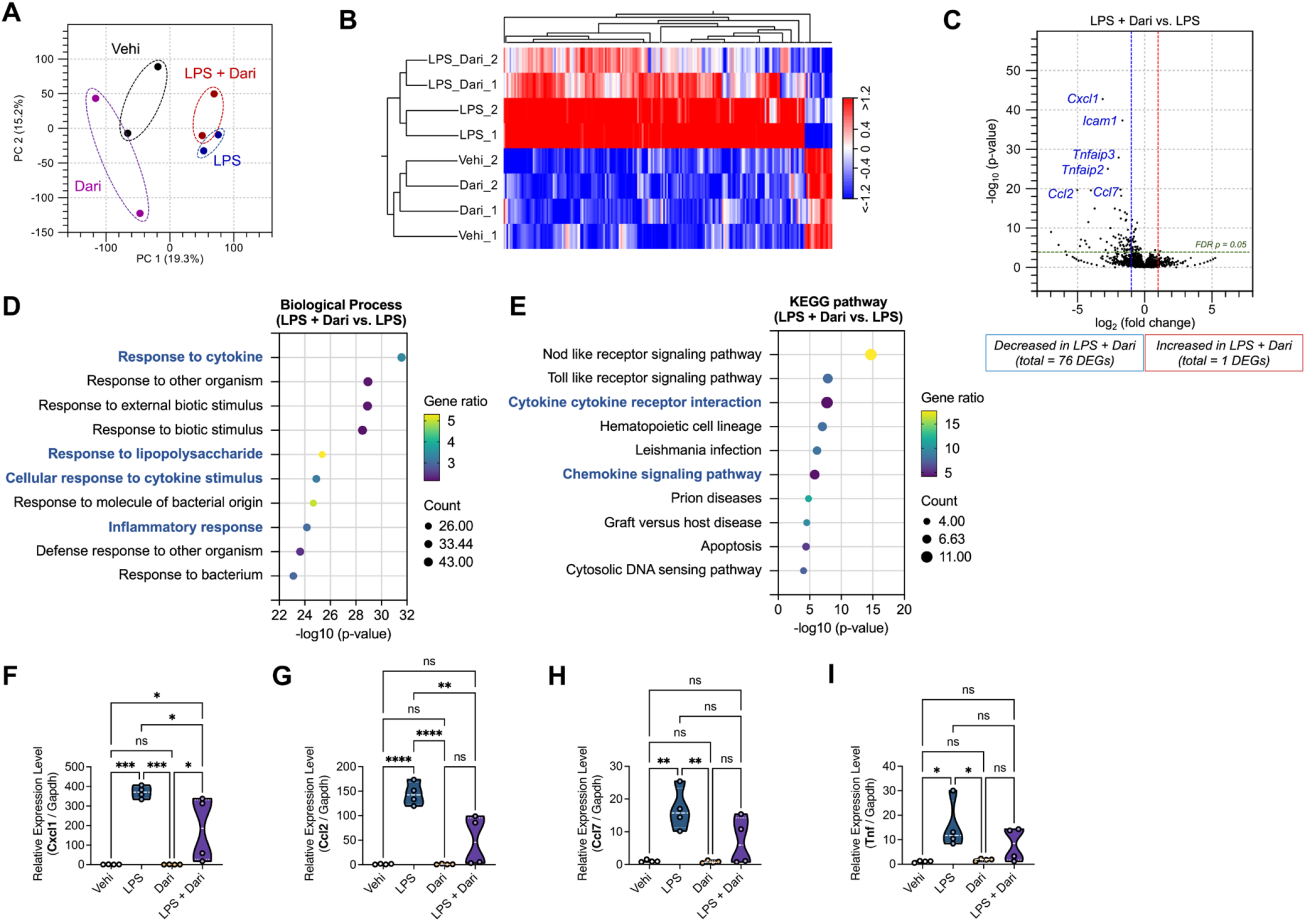
Comprehensive analysis of the hypothalamic transcriptome between the LPS and LPS + Daridorexant groups. (A) Principal component analysis (PCA) of the RNA‐seq data. Principal component 1 (PC1) (x‐axis) and PC2 (y‐axis) account for 19.3% and 15.2% of the variation, respectively, across Vehicle, Daridorexant, LPS, and LPS + Daridorexant groups. Each dot denotes a single biological replicate, and the dashed circles represent two replicates for each sample. Black dots, Vehicle group; magenta dots; Daridorexant group, blue dots; LPS group, and red dots; LPS + Daridorexant group. (B) Hierarchical clustering of the expression profiles among four groups. Individual samples are provided in columns and genes in rows. Heatmaps represent the relative expression (red; high, white; intermediate, blue; low expression). (C) Volcano plots represent the differentially expressed genes (DEGs) between the LPS and LPS + Daridorexant groups. Dotted vertical lines, log2 FC ≥ 2 or ≤ −2; dotted horizontal line, the significance cut‐off (false discovery rate: *P* = 0.05). (D, E) Functional enrichment analysis of 76 downregulated DEGs in the LPS + Dari group vs. LPS group, the negative log10 of the *p* value. The top 10 enriched gene ontology (GO) terms associated with biological process (D) and Kyoto Encyclopedia of Genes and Genomes (KEGG) pathway (https://www.kegg.jp/kegg/kegg1.html) analysis (E). (F–I) Relative expression of inflammation‐related genes in hypothalamic tissues, *Cxcl1* (F), *Ccl2* (G), *Ccl7* (H), and *Tnf* (I) normalized to *Gapdh* analyzed using qRT‐PCR. Data are presented as mean ± standard errors of the mean for *n* = 4 per group. *p < 0.05, **p < 0.01, ***p < 0.001, ****p < 0.0001, and ns (not significant). Statistical analysis was performed using one‐way analysis of variance followed by Tukey's post hoc test.

**FIGURE 4:**
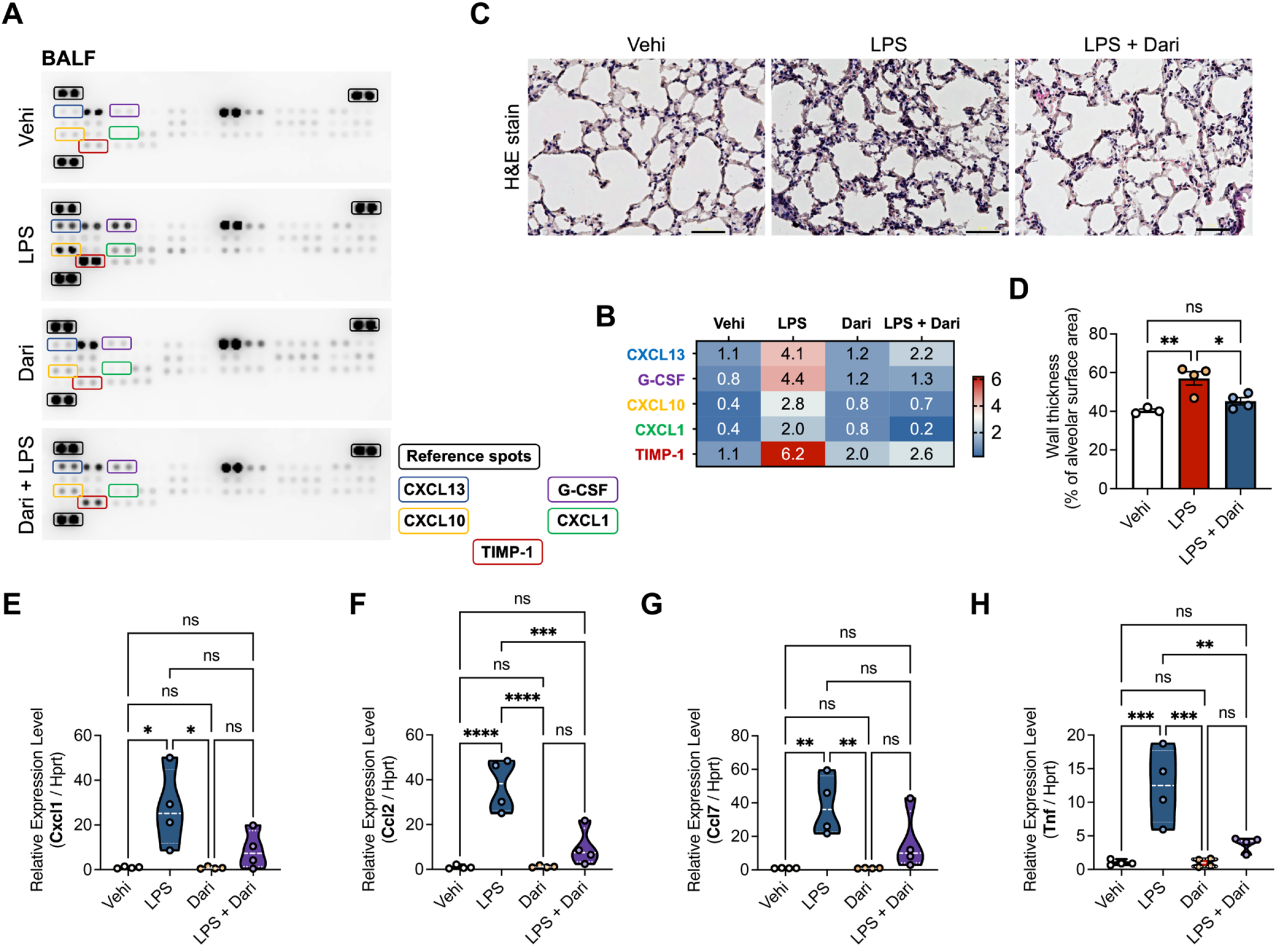
Protective effects of orexin receptor antagonism against acute lung injury in LPS‐induced inflammatory mice. (A) Typical expression profile of cytokines. The mouse cytokine array was used to determine the regulation of 40 proteins in BALFs of the Vehicle, Daridorexant, LPS, and LSP + Daridorexant groups (for the protein table, see www.RnDSystems.com). (B) Changes in expression of CXCL13, G‐CSF, CXCL10, CXCL1, and TIPM‐1 observed in western blot array analyzed by densitometer. Using a densitometer, each signal was normalized to the positive internal controls included in the array membrane and expressed in arbitrary units of 10. (C) Histological analysis of the lobe of left lungs. Representative photographs of hematoxylin and eosin (H&E) stains in the Vehicle, LPS, and LPS + Daridorexant groups (scale bars, 50 μm). (D) Quantitative analysis of alveolar wall thickness, expressed as a percentage of the alveolar surface area. Values were averaged across four randomly selected fields per section. (E–H) Relative expression of inflammation‐related genes in lung tissues, *Cxcl1* (E), *Ccl2* (F), *Ccl7* (G), and *Tnf* (H), normalized to *Hprt* analyzed using qRT‐PCR. Data are presented as mean ± standard errors of the mean for *n* = 4 per group. *p < 0.05, **p < 0.01, ***p < 0.001, ****p < 0.0001, and ns (not significant). Statistical analysis was performed using one‐way analysis of variance followed by Tukey's post hoc test.

Group differences were assessed using unpaired two‐tailed t‐tests or one‐way ANOVA, depending on the dataset. For EEG/EMG data, including sleep architecture parameters (percentages of wakefulness, NREM, and REM sleep), comparisons across groups and experimental days were conducted using one‐way ANOVA followed by Tukey's multiple comparison test (Figures 1 and 2). When baseline normalization was applied, data were expressed as a percentage of Day 1 values to account for inter‐individual variability. For sleep episode analyses (Figure 3), unpaired two‐tailed Student's t‐tests were applied. For qRT‐PCR results, one‐way ANOVA followed by Tukey's multiple comparison test was applied (Figures 3 and 4). For lung pathological analyses, including histological quantification (Figure 4), one‐way ANOVA followed by Tukey's post hoc test was used; in cases of two‐group comparisons, unpaired t‐tests were employed. When assumptions such as homogeneity of variance or normality were not met, more robust statistical tests were used, including Welch's t‐test, Welch's ANOVA, Mann–Whitney U test, or Kruskal–Wallis test with Dunn's correction. Post hoc analyses, including Tukey's or Bonferroni's tests, were performed to correct for multiple comparisons while controlling type I error. All values are presented as mean ± standard error of the mean (SEM) unless otherwise indicated. Exact *p* values and 95% confidence intervals are reported in the Results and figure legends for all major comparisons. A *p* value of < 0.05 was considered statistically significant. Statistical analyses for EEG/EMG, qRT‐PCR, and lung pathological data were performed using GraphPad Prism (version 9.1; GraphPad Software Inc., San Diego, CA, USA).

## 3 Results

### 3.1 Pretreatment With Daridorexant Alters Sleep Architecture in Mice With LPS‐Induced Inflammation

To evaluate the effects of orexin activity on the sleep architecture of LPS‐induced mice, we compared the sleep and wakefulness balance between vehicle and daridorexant groups. As shown in (Figure 1B–D), daridorexant did not alter the balance between wakefulness and sleep in the noninflammatory state. In contrast, LPS administration significantly increased the percentage of total time spent in NREM sleep while reducing REM sleep and wakefulness (NREM: Vehi 148.6 ± 24.9% vs. LPS 297.1 ± 20.27%, *p* < 0.01; REM: Vehi 244.3 ± 39.9% vs. LPS 1.2 ± 0.5%, *p* < 0.0001; WAKE: Vehi 79.5% ± 9.1% vs. LPS 11.4 ± 5.9%, *p* < 0.01). This indicated that LPS‐induced systemic inflammation alters sleep architecture by favoring NREM sleep at the expense of REM sleep and wakefulness. In the LPS‐induced inflammatory state, daridorexant decreased the percentage of total time spent in NREM sleep in the dark phase (ZT 12 to ZT 24) (LPS 297.1 ± 20.3% vs. LPS + Dari 185.9 ± 45.0%, 95% CI [10.6–211.9], *p* < 0.05). On the next recovery day, the time spent in wakefulness was significantly longer in the LPS + Daridorexant group than in the LPS group in the light phase (ZT 0 to ZT 12) (LPS 50.4 ± 13.9% vs. LPS + Dari 110.0 ± 4.7%, *p* < 0.05) (Figure 1A,B). As shown in Figure S1, LPS administration markedly increased total NREM sleep while reducing REM sleep and wakefulness (NREM: Vehi 285.1 ± 32.5 min vs. LPS 667.0 ± 25.2 min, *p* < 0.0001; REM: Vehi 49.8 ± 6.1 min vs. LPS 0.2 ± 0.06 min, *p* < 0.001; Wake: Vehi 396.2 ± 38.4 min vs. LPS 52.9 ± 25.2 min, *p* < 0.0001). In the LPS‐induced inflammatory state, daridorexant significantly reduced the total time spent in NREM sleep during the dark phase (ZT12–24) (LPS 667.0 ± 25.2 min vs. LPS + Dari 424.0 ± 108.8 min, *p* < 0.01). On the recovery day, wakefulness time in the dark phase (ZT12–18) was significantly greater in the LPS + Daridorexant group compared with the LPS group (LPS 103.9 ± 26.3 min vs. LPS + Dari 222.0 ± 31.4 min, *p* < 0.05). Taken together, these results suggest that the improvement of sleep architecture due to daridorexant administration would bring about changes in wakefulness on the next recovery day.

### 3.2 Orexin Receptor Antagonism Changes REM Episodes in Mice With LPS‐Induced Inflammation

During the dark period of the LPS injection day, the LPS + Daridorexant group exhibited a significant increase in the number of REM episodes compared to the LPS group (LPS 0.7 ± 0.3 vs. LPS + Dari 17.0 ± 8.4 episodes, *p* < 0.05) (Figure 2A). Additionally, the mean duration of REM episodes was markedly longer in the LPS + Daridorexant group than in the LPS group (LPS 3.3 ± 2.1 s vs. LPS + Dari 34.4 ± 20.8 s, *p* < 0.05). Conversely, the number of episodes and mean duration of NREM sleep did not differ significantly between the LPS and LPS + Daridorexant groups (*p* = n.s.) (Figure 2B). On the recovery day, the LPS + Daridorexant group exhibited recovery of wakefulness during the dark phase (ZT 12 to ZT 18), when mice were most active. Wakefulness levels reached 79.5% ± 8.4% (95% CI [1.9–75.3]) of those on the intact day. As a result of the improved sleep architecture on the LPS injection day, the LPS + Daridorexant group exhibited a significant increase in wakefulness on the recovery day compared to the LPS group (LPS 40.9% ± 11.6% vs. LPS + Dari 79.53% ± 8.4%, *p* < 0.05) (Figure 2C), nearly returning to the basal levels observed on the intact day.

### 3.3 Daridorexant Suppresses the Expression of Inflammation‐Associated Genes in the Hypothalamic Area of Mice With LPS‐Induced Inflammation

Sleep quality is critical in regulating systemic inflammation, and systemic inflammation, in turn, sends signals to the brain in response to infectious challenges [2, 17]. Given the significant effects of daridorexant on LPS‐induced alterations in sleep architecture, we investigated gene expression profiles in hypothalamic tissues using RNA‐seq analysis.

To assess the reproducibility of biological replicates between the Vehicle and LPS groups, we performed clustering using principal component analysis (PCA) and hierarchical clustering analysis. Both analyses revealed distinct separation between the two groups, indicating robust differences in gene expression profiles (Figure S1A,B). We identified 214 differentially expressed genes (DEGs) between the LPS and Vehicle groups, with 196 upregulated and 18 downregulated in the LPS group (Figure S1C). To characterize the functional features of these DEGs, we conducted gene ontology (GO) annotation and Kyoto Encyclopedia of Genes and Genomes (KEGG) pathway enrichment analyses (Table S2). The biological processes enriched in the DEGs included “response to cytokine”, “cellular response to cytokine stimulus”, and “response to lipopolysaccharide” (Figure S1D,E). KEGG pathway analysis further identified terms such as “cytokine‐cytokine receptor interaction”, “apoptosis”, and “chemokine signalling pathway” as significantly enriched. These results demonstrate that LPS injection upregulates inflammation‐associated genes in the hypothalamic tissues, contributing to systemic inflammation.

To elucidate the molecular mechanisms underlying the inhibitory effects of daridorexant on LPS‐induced inflammation, we analyzed gene expression profiles in hypothalamic tissues from the Vehicle, Daridorexant, LPS, and LPS + Daridorexant groups. Hierarchical clustering analysis revealed notable differences between the LPS and LPS + Daridorexant groups (Figure 3B), though the PCA score plot did not show distinct clusters (Figure 3A). Using differences in reads per kilobase of exon per million mapped reads, we identified 77 DEGs between the LPS and LPS + Daridorexant groups, including one upregulated and 76 downregulated genes (Figure 3C and Table S2). Notably, downregulated genes included *Cxcl1, Icam1, Ccl2, Ccl7, Tnfaip3*, and *Tnfaip2*, which are implicated in pro‐inflammatory pathways. GO and KEGG pathway analyses of the DEGs revealed enrichment in terms such as “response to cytokines”, “response to lipopolysaccharide”, “cellular response to cytokine stimulus”, and “inflammatory response”, along with pathways including “cytokine‐cytokine receptor interaction” and “chemokine signalling pathway” (Figure 3D,E). These results suggest that LPS‐induced inflammatory responses in the hypothalamus are attenuated by daridorexant treatment. To validate these results, we measured the expression of key proinflammatory cytokine genes (*Cxcl1, Ccl2, Ccl7*, and *Tnf*) using quantitative reverse transcription qRT‐PCR. Consistent with the RNA‐seq data, these genes were significantly upregulated in the LPS group compared to the Vehicle group. Pretreatment with daridorexant significantly reduced the expression of these genes in the LPS + Daridorexant group (Figure 3F–3I). These results further confirm that daridorexant mitigates LPS‐induced inflammatory responses by suppressing the expression of pro‐inflammatory genes in the hypothalamus.

### 3.4 Pretreatment With Daridorexant Protects Against LPS‐Induced Lung Inflammation

The LPS‐induced acute inflammatory response in mice is characterized by increased airway cell numbers and elevated levels of pro‐inflammatory cytokines and chemokines in the bronchoalveolar lavage fluid (BALF). These changes would lead to increased airway epithelium permeability and lung inflammation [18]. To investigate the effects of daridorexant on LPS‐induced inflammation, we measured the levels of pro‐inflammatory cytokines and chemokines in BALF samples from Vehicle, LPS, Daridorexant, and LPS + Daridorexant groups using a mouse cytokine array. The cytokines and chemokines CXCL1, CXCL10, CXCL13, G‐CSF, and TIMP‐1 were markedly increased in the LPS group compared to the Vehicle group. Notably, these levels were significantly reduced in the BALF of the LPS + Daridorexant group compared to the LPS group, indicating that daridorexant attenuates LPS‐induced inflammation in the lung (Figure 4A,B).

To further evaluate whether orexin receptor antagonism reduces inflammatory changes in lung tissues, we performed histological analysis using hematoxylin and eosin staining on lung samples from Vehicle, LPS, and LPS + Daridorexant groups. As shown in (Figure 4C,D), LPS treatment led to pronounced inflammatory cell infiltration and thickening of the alveolar wall, which were significantly reduced in the LPS + Daridorexant group.

To confirm the molecular mechanisms underlying this effect, we measured the mRNA expression of pro‐inflammatory genes *Cxcl1, Ccl2, Ccl7*, and *Tnf* using qRT‐PCR. Consistent with BALF and histological findings, the expression of these genes was significantly upregulated in the LPS‐group compared to the Vehicle group and was attenuated in the LPS + Daridorexant group (Figure 4E–H). Collectively, these data demonstrate that pretreatment with daridorexant reduces LPS‐induced lung inflammation by suppressing the expression of pro‐inflammatory cytokines and chemokines and alleviating histological damage.

## 4 Discussion

Sleep is an essential component of human health, with its timing, duration, and quality being critical determinants of overall well‐being [17]. In the present study, we demonstrated that orexin receptor antagonism improved sleep quality and suppressed pro‐inflammatory cytokine levels, leading to the mitigation of systemic inflammation in an LPS‐induced inflammatory model.

Orexin neuropeptides, such as orexin‐A and ‐B, are released by hypothalamus neurons and regulate physiological processes including appetite, arousal, energy homeostasis, and the reward system [9, 19]. These peptides bind to their respective receptors, orexin receptor type 1 (OX1R) and type 2 (OX2R) to activate signaling pathways that mediate these functions [20]. DORAs, such as daridorexant, block the activity of both OX1R and OX2R and induce a rapid transition to sleep [21, 22]. Under healthy conditions, a sleep episode begins with NREM sleep, and cycles between NREM and REM stages are maintained throughout the night, with REM sleep duration progressively increasing during later cycles [23, 24]. Our results show that daridorexant significantly increased the number and mean duration of REM episodes during the LPS‐exposed dark period (Figure 2B,C). In addition, this study indicated that daridorexant treatment altered the percentage of NREM sleep toward that of the normal state, suggesting that in the acute inflammatory state, daridorexant treatment reverses the sleep architecture toward that of the normal state. However, daridorexant administration during the light phase did not alter sleep architecture when natural sleep patterns were maintained in healthy mice (Figure 1). Because rodents exhibit phase‐dependent sleep–wake regulation, with the light phase as the primary rest period and the dark phase as the active period [25, 26], we analyzed both phases to capture the biological significance of phase‐specific modulation under systemic inflammation. This observation suggests that orexin activity may naturally be lower during the light phase, limiting the effects of its antagonism on sleep.

Acute sleep loss is closely linked to the release of inflammatory mediators, and poor sleep quality is positively associated with increased levels of proinflammatory cytokines, including IL6, IL10, CRP, TNF‐α, CXCL1, CXCL2, and IFN‐γ [27]. LPS‐stimulated neutrophils co‐localize in the brain with ICAM1‐positive cells, and related gene expressions of inflammatory markers such as IL6, CXCL1, and ICAM1 are observed in the hypothalamus, linking peripheral cytokine levels (e.g., IL6 and TNF‐α) to central neural inflammation [28]. Consistent with these findings, our transcriptomic analysis revealed that LPS upregulated cytokine and chemokine genes in hypothalamic tissues, including Icam1, Gbp5, Akap12, Adamts1, Ptgs2, Cxcl1, and Ifit1 (Figure S1). Pretreatment with daridorexant significantly downregulated Cxcl1 expression, suggesting that CXCL1, a neutrophil‐specific chemokine, plays a critical role in LPS‐induced inflammation (Figure 3C,F). These results indicate that daridorexant mitigates hypothalamic inflammation, improves wakefulness and REM sleep, and may be effective in improving sleep–wake cycles in the context of systemic inflammation.

Sleep is a well‐recognized regulator of immune responses [2, 17], and its disruption has been implicated in the exacerbation of systemic inflammation, including lung inflammation [29, 30]. Previous research has emphasized the importance of regular sleep patterns for reducing lung inflammation [31]. In our study, daridorexant improved LPS‐induced lung pathology, as evidenced by reduced inflammatory chemokine levels and improved lung histology (Figure 4). Although the reduction in inflammatory markers, such as CXCL1 and CCL2, was observed in both hypothalamic and lung tissues, whether this effect was directly mediated in the lungs or indirectly through the suppression of hypothalamic neural inflammation remains unclear. This question warrants further investigation to elucidate the pathways involved.

Although our findings highlight the therapeutic potential of daridorexant, there are limitations that should be considered. The reliance on a mouse model limits the direct applicability of these results to humans. Additionally, the long‐term effects of orexin receptor antagonism on sleep and inflammation remain unknown.

Importantly, the dose of daridorexant used in mice (108 mg/kg) was considerably higher than the approved clinical dose in humans (25–50 mg/day; 0.36–0.71 mg/kg for a 70‐kg adult). This discrepancy is primarily attributable to species differences in pharmacokinetics and drug metabolism, as rodents generally require higher mg/kg doses to achieve comparable receptor occupancy and pharmacodynamic effects. Consistent with this notion, a previous nonclinical study demonstrated that daridorexant produced robust sleep‐promoting effects in rodents at dose levels up to 300 mg/kg while maintaining physiological sleep architecture, with no evidence of tolerance development or physical dependence during repeated administration [12]. Therefore, the present work should be regarded as a mechanistic proof‐of‐concept study rather than a direct translation of human dosing. Furthermore, accumulating evidence suggests that dual orexin receptor antagonists (DORAs) may exert not only hypnotic effects but also broader modulatory influences on inflammatory and neurobiological processes. Recent preclinical studies have shown that pharmacological inhibition of orexin signaling can affect immune‐ and glia‐related pathways across both peripheral and central tissues, contributing to improved tissue homeostasis in various experimental settings [32, 33]. These observations add translational relevance to our findings and imply that DORAs may hold broader therapeutic potential in inflammatory and neurodegenerative conditions. In line with these reports, our present findings indicate that orexin receptor antagonism acted protectively under systemic inflammatory conditions by promoting restorative sleep and attenuating excessive inflammation.

In the present study, we used male mice to minimize biological variability and ensure experimental consistency. Previous studies have reported sex‐related differences in sleep architecture and responses to stressors such as sleep deprivation or restraint stress [34]. Therefore, future investigations including female mice will be valuable to clarify sex‐specific effects and strengthen the translational relevance of orexin‐related mechanisms. Further studies should explore the translational potential of these findings, including clinical trials to evaluate daridorexant's efficacy in inflammatory conditions with disrupted sleep–wake cycles.

This study underscores the potential of targeting orexin signaling to modulate systemic inflammation and improve sleep quality. The findings pave the way for developing therapeutic strategies for inflammatory diseases characterized by sleep disorders. Future research should focus on validating these results in human populations, investigating the long‐term effects of DORAs, and elucidating the molecular mechanisms underlying their central and peripheral actions.

## Author Contributions

D.H., Y.I.‐T, J‐.D.K., and K.T. were involved in the study design and conceptualization. D.H., Y.I.‐T, and J‐.D.K. were involved in the animal and laboratory experiments. D.H., Y.I.‐T, J‐.D.K., Y.K., and K.T. wrote the manuscript. T.N., Y.N., and T.S. supervised the study. All authors approved the final version of the manuscript.

## Funding

This work was supported by MEXT JSPS Japan Society for the Promotion of Science London (JSPS) (24K11336, 24K08827) and Japan Agency for Medical Research and Development (AMED) (JP21gm1410010).

## Conflicts of Interest

The authors declare no conflicts of interest.

## Supporting information


**Figure S1:** fsb271408‐sup‐0001‐Figures.pdf.
**Figure S2:** fsb271408‐sup‐0001‐Figures.pdf.

**Table S1:** The list of the upregulated (left) or downregulated (right) DEGs from the RNA‐seq analysis between control (Vehi) and LPS groups.
**Table S2:** The list of the upregulated (left) or downregulated (right) DEGs between LPS and LPS + Dari groups.

## Data Availability

All data required to evaluate the conclusions of the paper are presented in this study or upon reasonable request from the corresponding authors. All raw data files for the RNA‐seq analysis were deposited in the NCBI for Biotechnology Information Gene Expression Omnibus (GEO) database (https://www.ncbi.nlm.nih.gov/geo; accession number: GSE281503).
