# Supplementary material for: Orexin Receptor Antagonism Improves Sleep Quality and Mitigates Lipopolysaccharide‐Induced Inflammatory Responses in a Mouse Model
Source: FASEB J. 2026 Jan 9;40(1):e71408. doi: 10.1096/fj.202502960R (PMC12785484; doi:10.1096/fj.202502960R)
Supplement: Supplementary file 1 — Figure S1: fsb271408‐sup‐0001‐Figures.pdf. Figure S2: fsb271408‐sup‐0001‐Figures.pdf. [file FSB2-40-e71408-s002.pdf]

Supplementary Information

Orexin receptor antagonism improves sleep quality and mitigates lipopolysaccharide-induced inflammatory responses in mice

Dai Horiuchi, Yoko Irukayama-Tomobe, Jun-Dal Kim[#], Yoshitoshi Kasuya, Tsuyoshi Nemoto, Takuji Suzuki, Yoshimi Nakagawa, Koichiro Tatsumi[#]

[#]**Correspondence**: Jun-Dal Kim (jdkim@inm.u-toyama.ac.jp); Koichiro Tatsumi (tatsumi@faculty.chiba-u.jp)

Supplementary Figures S1-S2
Supplementary Tables S1-S2 (1 excel file)

Figure S1

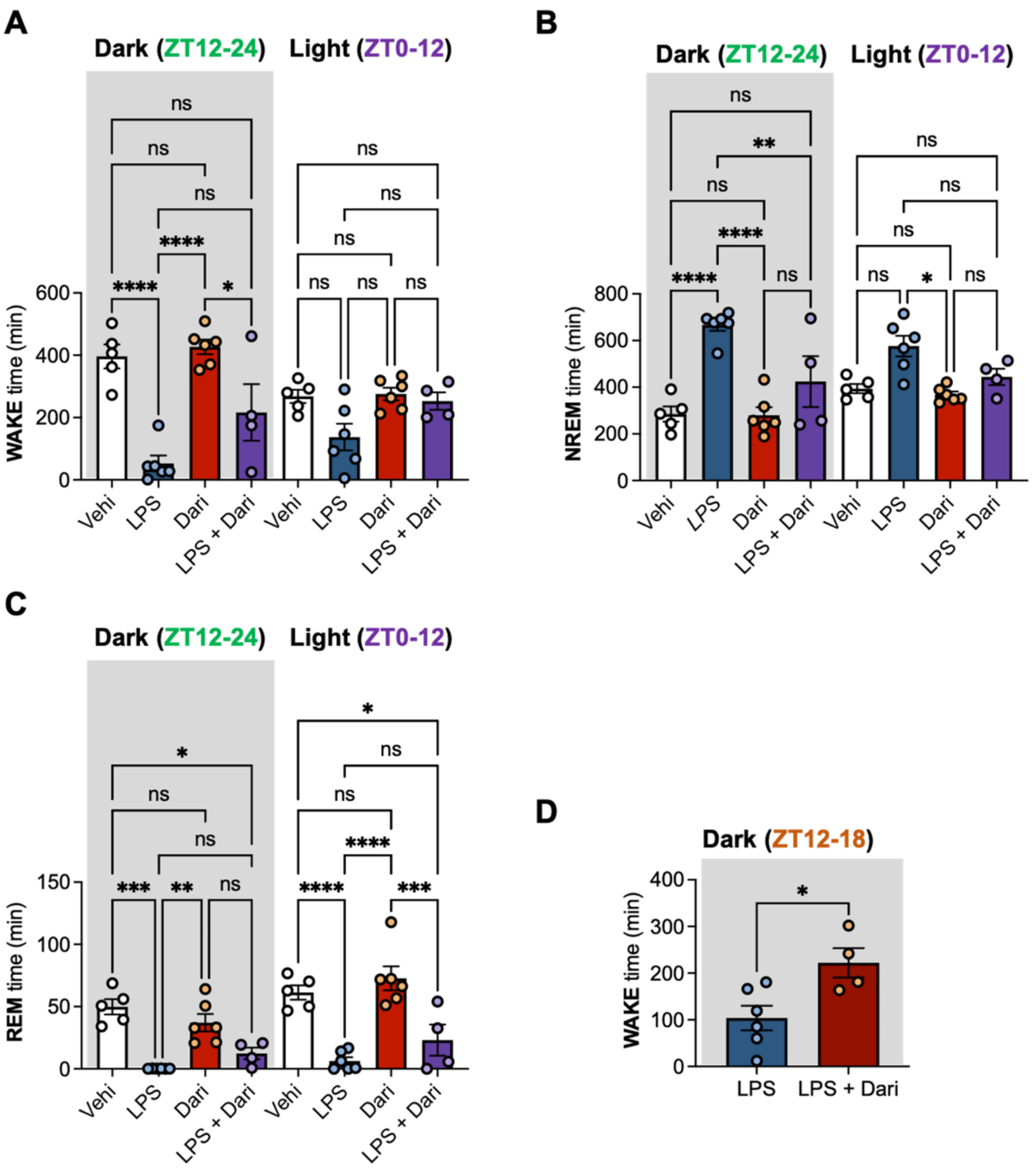

Figure S1. Total time spent per state (Wake/NREM/REM) under LPS challenge and daridorexant treatment. Effects of LPS and/or daridorexant administration on wakefulness (**A**), NREM sleep (**B**), and REM sleep (**C**) during the dark phase (ZT12–24). (**D**) Wakefulness time during the dark phase (ZT12–18) on the recovery day (Day 5). Vehi = Vehicle; Dari = Daridorexant; LPS + Dari = LPS + Daridorexant. Data are shown as mean ± SEM (n = 4–6 per group). *p < 0.05, **p < 0.01, ***p < 0.001, ****p < 0.0001, ns = not significant. Statistical analyses were performed using one-way ANOVA followed by Tukey's post hoc test for multiple comparisons (A–**C**). For panel (D), unpaired two-tailed Student's t-tests were applied.

Figure S2

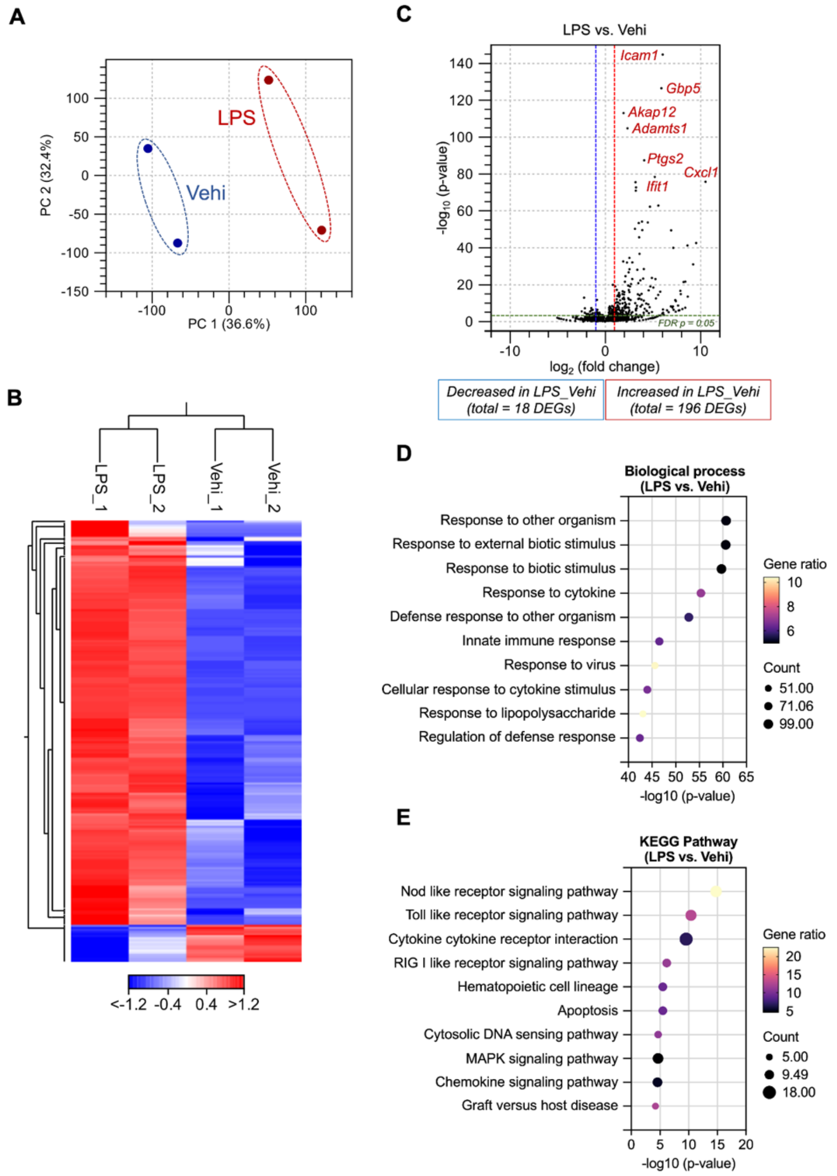

Figure S2. Comprehensive analysis of the hypothalamic transcriptome between the Vehicle and LPS groups. (**A**) Principal component analysis (PCA) of the RNA-seq data. Principal component 1 (PC1) (x-axis) and PC2 (y-axis) explain 36.6% and 32.4% of the variation, respectively, between Vehicle and LPS groups. Each dot denotes a single biological replicate, and the dashed circles represent two replicates for each sample. Blue dots; Vehicle group and red dots; LPS group. (**B**) Hierarchical clustering of the expression profiles between two groups. Individual samples are provided in columns and genes in rows. Heatmaps represent the relative expression (red; high, white; intermediate, blue; low expression). (**C**) Volcano plots represent the differentially expressed genes (DEGs) between the Vehicle and LPS groups. Dotted vertical lines, log2 FC ≥ 2 or ≤ -2; dotted horizontal line, the significance cut-off (false discovery rate: $p = 0.05$). (**D**, **E**) Functional enrichment analysis of 196 upregulated DEGs in the LPS group vs. Vehicle group, the negative log10 of the *p*-value. The top 10 enriched gene ontology (GO) terms associated with biological process (D) and Kyoto Encyclopedia of Genes and Genomes (KEGG) pathway (https://www.kegg.jp/kegg/kegg1.html) analysis (E).

Supplementary Table S1. The list of the upregulated (left) or downregulated (right) DEGs from the RNA-seq analysis between control (Vehi) and LPS groups.
(Excel file)

Supplementary Table S2. The list of the upregulated (left) or downregulated (right) DEGs between LPS and LPS+Dari groups.
(Excel file)
